# Complex Human Activity Recognition Using Smartphone and Wrist-Worn Motion Sensors

**DOI:** 10.3390/s16040426

**Published:** 2016-03-24

**Authors:** Muhammad Shoaib, Stephan Bosch, Ozlem Durmaz Incel, Hans Scholten, Paul J. M. Havinga

**Affiliations:** 1 Pervasive Systems Group, Department of Computer Science, Zilverling Building, PO-Box 217, 7500 AE Enschede, The Netherlands; stephan@inertia-technology.com (S.B.); hans.scholten@utwente.nl (H.S.); p.j.m.havinga@utwente.nl (P.J.M.H.); 2 Department of Computer Engineering, Galatasaray University, Ortakoy, 34349 Istanbul, Turkey; odincel@gsu.edu.tr

**Keywords:** body-worn sensing, behavior analysis, sensor fusion, gesture recognition, smartwatch sensors, smoking recognition

## Abstract

The position of on-body motion sensors plays an important role in human activity recognition. Most often, mobile phone sensors at the trouser pocket or an equivalent position are used for this purpose. However, this position is not suitable for recognizing activities that involve hand gestures, such as smoking, eating, drinking coffee and giving a talk. To recognize such activities, wrist-worn motion sensors are used. However, these two positions are mainly used in isolation. To use richer context information, we evaluate three motion sensors (accelerometer, gyroscope and linear acceleration sensor) at both wrist and pocket positions. Using three classifiers, we show that the combination of these two positions outperforms the wrist position alone, mainly at smaller segmentation windows. Another problem is that less-repetitive activities, such as smoking, eating, giving a talk and drinking coffee, cannot be recognized easily at smaller segmentation windows unlike repetitive activities, like walking, jogging and biking. For this purpose, we evaluate the effect of seven window sizes (2–30 s) on thirteen activities and show how increasing window size affects these various activities in different ways. We also propose various optimizations to further improve the recognition of these activities. For reproducibility, we make our dataset publicly available.

## 1. Introduction

On-body motion sensors are commonly used in recognizing various activities. For example, mobile phone motion sensors have been a popular choice for activity recognition at the trouser pocket or equivalent position (referred to as the pocket in the rest of the paper) [1,2]. Recently, wrist-worn motion sensors are also being used for human activity recognition [3,4,5]. The position of these sensors plays an important role in such recognition. For example, some activities, such as smoking, eating, writing, typing, drinking coffee and giving a talk, cannot be recognized reliably at the pocket position, because these mainly involve hand movements. However, these activities can be recognized with wrist-worn motion sensors. Other activities can be recognized easily by motion sensors at the pocket position, such as biking, walking upstairs and walking downstairs. In such activities, there is a better repetitive pattern of motion data at the pocket position than at the wrist position. However, these two positions have been mainly used in isolation for recognizing various activities [6,7,8,9]. To use the richer context information from both wrist and pocket, we evaluated the effect of using motion sensors at both of these positions with respect to the wrist position alone.

For this purpose, we recognized thirteen activities, which we divide into two groups: simple and complex activities. Simple activities are repetitive in nature and can be easily recognized using an accelerometer at the pocket and wrist position; for example, walking, jogging, biking, writing, typing, sitting and standing. Complex activities are not as repetitive as simple activities and may involve various hand gestures; for example, eating, drinking coffee, smoking and giving a talk. We also place using stairs in this category, because it is not easy to recognize such activities with a single accelerometer. Usually, additional sensors, such as a gyroscope [10] and a barometer [11], are combined with the accelerometer to reliably recognize using stairs: walking upstairs and downstairs.

Besides combining motion sensors, the size of the segmentation window also affects the recognition of these activities. For example, a smaller window size of 2–5 s is usually sufficient for the reliable recognition of simple activities, such as walking, jogging and biking [10,12]. However, such a window size may not be enough for some complex (less-repetitive) activities, such as smoking, eating, giving a talk and drinking coffee, because we cannot obtain their proper repetitive pattern in a such a small window. Though the effect of window size on simple physical activities has been studied before [13], its effect on complex activities is yet to be explored. To see the effect of increasing window size on various activities, we evaluated the effect of seven window sizes on thirteen activities in various scenarios and showed that it affects simple and complex activities in different ways.

The reliable recognition of these activities can enable various well-being applications for detecting bad habits and providing context-aware feedback [14]. According to the World Health Organization, smoking, poor nutrition, harmful use of alcohol and physical inactivity are the main reasons for premature deaths [15]. These bad behaviors can be detected by recognizing activities, such as lack of physical activity, drinking, smoking, physical (in)activity and missing meals or not taking meals on time. Moreover, the recognition of such activities can be used in giving context-aware feedback [14]. For example, a user should not be interrupted for a feedback message while typing, writing or giving a talk, but can be interrupted while having a cup of coffee or smoking. Earlier studies [5,7,9,16,17,18] have shown that such activities can be recognized using on-body motion sensors, which we discuss further in the Related Work Section 2. However, these studies have used the wrist and pocket positions in isolation. Furthermore, unlike our work, these studies either consider many simple activities or one complex activity. For example, in [7], the authors focus on the eating activity only. Similarly, in [18], only the smoking activity is considered. Moreover, these studies did not explore the effect of increasing segmentation window size on these activities. They have mainly focused on the accelerometer and gyroscope, whereas we also evaluate the role of linear acceleration. We extended the existing work by investigating the following research questions: (1) How is the recognition performance of various human activities affected by combining motion sensors from both pocket and wrist positions with respect to the wrist position alone? (2) How does the size of the segmentation window affect the recognition performance of various simple and complex activities? Our main contributions are as follows:

- Using three classifiers, we evaluated three motion sensors at the wrist and pocket positions in various scenarios and showed how these sensors behave in recognizing simple and complex activities, when used at either position or both positions. We showed the relationship between the recognition performance of various activities with these sensors and positions (pocket and wrist).
- Using three classifiers, we evaluated the effect of increasing the window size for each activity in various scenarios and showed that increasing the window size (from 2–30 s) affects the recognition of complex and simple activities in a different way.
- We proposed optimizing the recognition performance in different scenarios with low recognition performance. Moreover, we made our dataset publicly available for reproducibility.

The rest of the paper is organized as follows. We describe related work in Section 2. In Section 3, we discuss the data collection and experimental setup. In Section 4, we discuss our results, limitations of this work and future work. Finally, in Section 5, we present the conclusions.

## 2. Related Work

Activity recognition using body-worn motion sensors in general [19] and especially using smartphone sensors has been studied in recent years [1,2], and it is still being studied extensively [1,2,19,20,21]. There are also a few studies on activity recognition using wrist-worn devices. For example, in [6], the authors studied the role of smartwatch and smartphone sensors in activity recognition. They recognized nine physical activities using five classifiers. These activities were sitting, standing, walking, running, cycling, stair descent, stair ascent, elevator descent and elevator ascent. However, the authors studied these two devices separately and did not combine sensor data from both of these devices. They used accelerometer, magnetometer, gyroscope and pressure sensors on the smartphone and only an accelerometer on the smartwatch.

In [5], the authors used a wrist-worn sensor and a sensor on the hip to detect seven physical activities. They used logistic regression as a classifier. They showed the potential of using the wrist position for activity recognition. However, they evaluated these two positions separately and did not combine these two sensors. In [16], the authors use a single wrist-worn accelerometer to detect five physical activities. These activities were sitting, standing, lying, walking and running. Similarly, a wrist-worn accelerometer was used in [17] to recognize eight activities, including the activity of working on a computer.

In [7], the authors detect eating activity using a Hidden Markov Model (HMM) with a wrist-worn accelerometer and a gyroscope. They recognize eating by dividing that activity into sub-activities: resting, eating, drinking, using utensil and others. The authors report an accuracy of 84.3%. Similarly, in [9], the authors recognize the eating activity using a wrist-worn accelerometer and a gyroscope. They differentiate eating periods from non-eating periods and report an accuracy of 81%. In [22], the authors use the accelerometer and gyroscope data from a smartwatch to recognize eating episodes from similar non-eating activities. Additionally, they investigate the smartwatch’s camera to take images of the food being consumed to analyze what a user is eating. A feasibility study on smoking detection using a wrist-worn accelerometer is done in [18], where the authors reported a user-specific accuracy of 70% for this activity. This study used only an accelerometer at the wrist position. The authors in [8] use an accelerometer, a gyroscope and a magnetometer at the wrist position to recognize smoking puffs. However, they only differentiate smoking from other activities. A similar work to our study is done in [23]; however, the authors in this study use SVM to recognize only six activities, such as walking, standing, writing, smoking, jacks and jogging. Moreover, they utilize the wrist and foot position unlike our work. We use the pocket position, which can be considered more comfortable and/or practical than the foot position.

Though various activities can be recognized using motion sensors at the wrist position, the pocket position can provide additional information that can improve the recognition of these activities. For example, biking and using stairs can be better recognized using the pocket position, whereas smoking and eating using the wrist position. Therefore, to extend the existing work, we evaluate the combination of the wrist and pocket positions for the reliable recognition of various activities. We also evaluate three motion sensors at these two positions alone and in combination. Moreover, unlike the existing work, we evaluate the effect of window size on each activity and its impact on the recognition performance of simple and relatively complex activities. Though the effect of window size on simple physical activities has been studied before [13], its effect on complex activities is yet to be explored. Finally, we suggest optimizations to improve the recognition of various activities.

We had previously performed a preliminary work [24] towards complex activity recognition using wrist and pocket positions. In this work, we evaluated the effect of combining accelerometer and gyroscope from both pocket and wrist position at a window of 2 s only. Moreover, we analyzed the effect of the window size on the gyroscope only for an increasing window of 2–10 s. We studied the effect of synchronization delay between smartwatch and smartphone on recognition performance. We also analyzed the effect of various sampling rates on the recognition performance in different scenarios. However, our previous work had still room for further improvements. For example, our dataset was not balanced for all thirteen activities, which can lead to biased results towards majority classes. Moreover, we used 50% overlap in segmenting raw data, which can lead to higher recognition performance. As data are randomized before stratified cross-validation in the WEKA (Waikato Environment for Knowledge Analysis) tool [25], overlap in the data can cause using some data in both training and testing, which can lead to higher recognition performance. We also did not consider the linear acceleration sensor in our previous work. In this work, we collected additional data and addressed all of these shortcomings besides extending it further, as discussed in Section 1. For example, we evaluated the effect of increasing the window size of 2–30 s on various activities. We analyzed the combination of different sensors in more detail. We considered the linear acceleration sensor and its combination with the gyroscope. Moreover, we proposed some methods to further improve the recognition of different activities that have low recognition performance.

## 3. Data Collection and Experimental Setup

We collected a dataset for thirteen human activities. We selected these activities because they can be used for detecting bad habits and for better context-aware feedback, as discussed in Section 1. Ten healthy male participants (age range: 23–35) took part in our data collection experiments. However, not all activities were performed by each participant. Seven activities were performed by all ten participants, which are walking, jogging, biking, walking upstairs, walking downstairs, sitting and standing. These activities were performed for 3 min by each participant. Seven out of these ten participants performed eating, typing, writing, drinking coffee and giving a talk. These activities were performed for 5–6 min. Smoking was performed by six out of these ten participants, where each of them smoked one cigarette. Only six participants were smokers among the ten participants. We used 30 min of data for each activity with an equal amount of data from each participant. This resulted in a dataset of 390 (13 × 30) min.

The activities were performed indoors, except biking and smoking. Using stairs was performed in a building with five floors at our university. While sitting and standing, the participants respectively sat and stood still alone without talking or doing anything else. For typing and writing, they typed some text on their laptops and wrote the same text on an A4 size paper. They drank a cup of coffee while sitting in our office lounge. For the presentation talk, they talked about their research topic in our meeting room. They smoked one cigarette while standing alone outside. The participants ate soup or yogurt while sitting in the lunch cafe. They used a spoon for eating, while the soup cup was on a table. All participants performed these activities alone and in a controlled environment. Doing these activities in groups or doing more than one of them at the same time may result in a different motion pattern. We intend to explore such different variations of these activities in future studies, where we will collect more data.

During this data collection, all participants carried two mobile phones (Samsung Galaxy S2 [26]) in their right pocket and at their right wrist. A smartwatch or wrist-worn device is emulated by using a smartphone at the wrist position. There are also other positions, such as hip, or upper arm, or a bag, where these devices can be used. However, we selected pocket and wrist, as these are the commonly-used positions for a mobile phone and smartwatch. The orientation of mobile phones was portrait with their screen pointing towards the body. We collected data at 50 samples per second from the phone’s accelerometer, its (virtual) linear acceleration sensor and its gyroscope. Linear acceleration is obtained by removing acceleration due to gravity from the accelerometer measurements. The acceleration due to gravity is especially useful for differentiating different static postures, such as sitting and standing [27]. However, it is also less generic in terms of classification, because it is more sensitive to changes in sensor orientation and body position [27,28]. For example, if a classifier is trained using an accelerometer for smoking while standing, it may not recognize smoking while sitting with the same recognition performance. However, these two variations of smoking can be recognized in a better way by a linear acceleration sensor, because static postures have almost the same linear acceleration. Unfortunately, due to this fact, the linear acceleration sensor cannot differentiate between sitting and standing. If sitting and standing are not important for an application, it may be a good idea to use a combination of the linear acceleration and gyroscope.

One of the participants was left-handed, so we placed one mobile phone on his left wrist, while we still placed the second one in his right pocket. For consistency with other participants, we changed the sign of one of the axes of these sensors, so that the data from the left-handed participant looks as though he had performed these activities using his right hand. For the rest of the participants, it was always the right pocket and the right wrist position. For data collection, we used our Android application [10,12]. We will publish this dataset on our website [29].

In the pre-processing phase, we extracted two time-domain features for these sensors: mean and standard deviation. We selected these two features because they have low complexity and have been shown to provide reasonable recognition accuracy for various activities [12,30]. Moreover, we did not want to use a more complex feature set blindly before evaluating a viable simple one. These features were extracted over a window of 2, 5, 10, 15, 20, 25 and 30 s with no overlap. The orientation of motion sensors can affect the recognition performance of various activities [31]. To counter such effects, we use the magnitude of these sensors as an extra dimension besides the *x*, *y* and *z* axes. This method has shown to be effective in other studies [31,32,33]. Then, the mean and standard deviation are extracted for all four dimensions of each sensor. For some specific optimizations, as discussed in Section 4.4, we also extracted five additional features: minimum, maximum, semi-quartile, median and the sum of the first ten FFT (Fast Fourier Transform) coefficients. We selected these features based on the literature study [30] and on the manual analysis of our raw data. As discussed in Section 4.4, these features provide additional information about various activities and are suitable for running on mobile phones because of their low complexities [30].

For performance analysis, we used Scikit-learn (Version 0.17), which is a Python-based machine learning toolkit [34]. We selected three classifiers, which are commonly used for practical activity recognition: Naive Bayes, k-nearest neighbor (KNN) and decision tree [35,36]. They are suitable for running on mobile phones with reasonable recognition performance [35,36]. They are implemented on mobile phones in various studies; *i.e.*, Naive Bayes in [37,38,39], decision tree in [40,41,42] and KNN in [31,32,39,43]. To make this work easily reproducible, we use these classifiers in their default mode with very few changes. For KNN, we use three nearest neighbors (*K* = 3), which counters the effects of noise and helps with avoiding a tie in majority voting. Moreover, we use the ball tree algorithm in KNN as a nearest neighbor algorithm. It is an efficient way to find the nearest neighbor without going through the whole dataset (brute force approach) [34]. We use decision tree in its default mode. For the decision tree, Scikit-learn uses an optimized version of the CART (Classification and Regression Trees) algorithm [34]. The parameter settings for these three classifiers are given in Appendix A1.

For performance evaluation, we used 10-fold stratified cross-validation. In this validation method, the whole dataset is divided into ten equal parts or subsets. In each iteration, nine of these parts are used for training purpose and one for testing. This process is repeated ten times, thereby using all data for training, as well as testing. Stratified means that each fold or part has the right proportion of each class. Though we analyzed the classification accuracy, precision and F-measure as performance metrics, we only present the F-measure results, because it incorporates both accuracy and precision. Since we are only interested in the relative comparison of different scenarios, the F-measure as a performance metric is sufficient for this purpose. Moreover, our reported observations in the F-measure are similar to those in accuracy and precision. In Scikit-learn stratified cross-validation can be done in two ways: with shuffling and without shuffling the dataset.

- With shuffling: In this method, we shuffle the data before they are divided into ten equal parts. This means that for each participant, some part of his or her data is used in training and the other part in testing. There is no overlap between training and testing data. In this case, the classification performance will be slightly higher and may be closer to a person-dependent validation method.
- Without shuffling: In this method, no shuffling is performed before dividing the whole data into ten equal parts. The order of the data is preserved. In this way, the classification performance will be slightly lower than the shuffling method. In our case, it resembles a person-independent validation for the seven activities that were performed by all ten participants. However, for the rest of the activities, it is not person independent. As the number of participants is less than 10, when we divide their data into ten equal parts, each part may contain data from more than one participant. This can lead to using data from one participant in both training and testing, with no overlap in data between training and testing sets. As the order of the time series data is preserved, the results are closer to the real-life situations.

Though we use these two methods, we only present in detail the results for the cross-validation without shuffling, which is more close to a realistic scenario. All of the results presented in the next sections are for cross-validation without shuffling, unless otherwise specified. We briefly discuss the results for the shuffling method in Section 4.3.

## 4. Results and Discussion

We evaluated three motion sensors for recognizing thirteen activities in various scenarios. At the wrist position, we first evaluated each sensor alone ($W_{A}$, $W_{G}$, $W_{L}$) and then in combination with other sensors ($W_{AG}$, $W_{LG}$). Finally, we evaluated the effect of combining these sensors at both the wrist and pocket positions ($WP_{AG}$, $WP_{LG}$, $WP_{A}$). We use these short notations in the remainder of the paper, where *W* stands for wrist, *P* for pocket, WP for both wrist and pocket positions, *A* for accelerometer, *G* for gyroscope and *L* for linear acceleration sensor. In all of these sensor combinations, we concatenated the features of individual sensors as input for a classifier. For example, in $WP_{AG}$, we concatenated 32 (8 × 4 = 32) features, where each sensor at either position has eight features (mean and standard deviation). For simplicity, we present the classification results of the Naive Bayes classifier in the main paper. The results for the KNN and decision tree classifiers are given in Appendix A. In the next subsections, we discuss the effects of combining motion sensors, window size and various optimizations on the recognition performance of our thirteen activities.

### 4.1. The Effect of Wrist and Pocket Combination on Recognition Performance

Extending our previous work [24], we evaluated the effect of combining motion sensors at the wrist and pocket positions, such as an accelerometer, a gyroscope and a linear acceleration sensor. For a simpler presentation of our results, we used the F-measure at $W_{AG}$ (accelerometer + gyroscope at wrist) and $W_{LG}$ (linear acceleration sensor + gyroscope at wrist) as the reference value. With respect to this reference value, we showed the effect of combining these sensors from both pocket and wrist positions in terms of performance increase, decrease or no change. Moreover, with such a presentation, we also showed how much $W_{AG}$ and $W_{LG}$ improve the recognition performance compared to when these sensors are used alone at the wrist ($W_{A}$, $W_{G}$, $W_{L}$). We chose the wrist position as a reference point, because all thirteen activities can be detected reliably using the wrist, unlike the pocket position. For example, it is not feasible to recognize activities involving hand gestures using the pocket position alone, such as eating and smoking.

**Figure 1:**
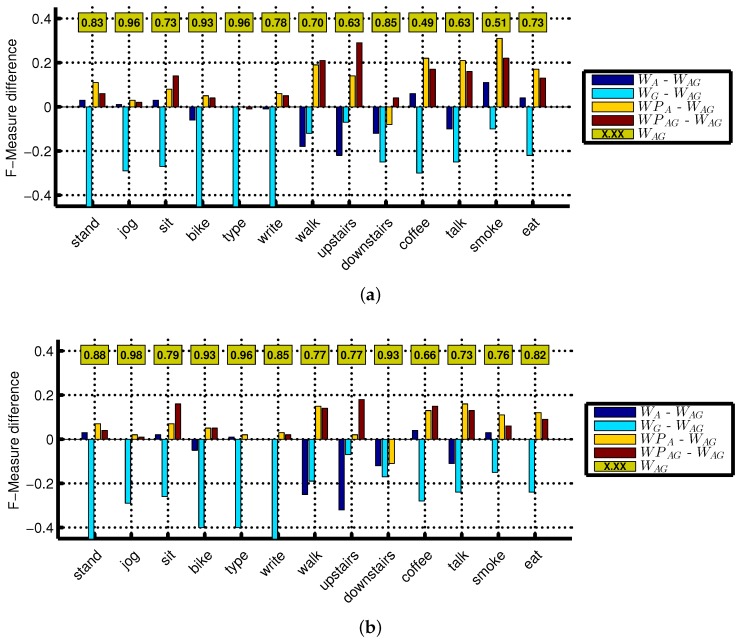
F-measure difference with respect to the reference ($W_{AG}$) for all activities using Naive Bayes classifier: (**a**) 2-s window; (**b**) 5-s window. The reference ($W_{AG}$) F-measure is shown in the boxes.

**Figure 2:**
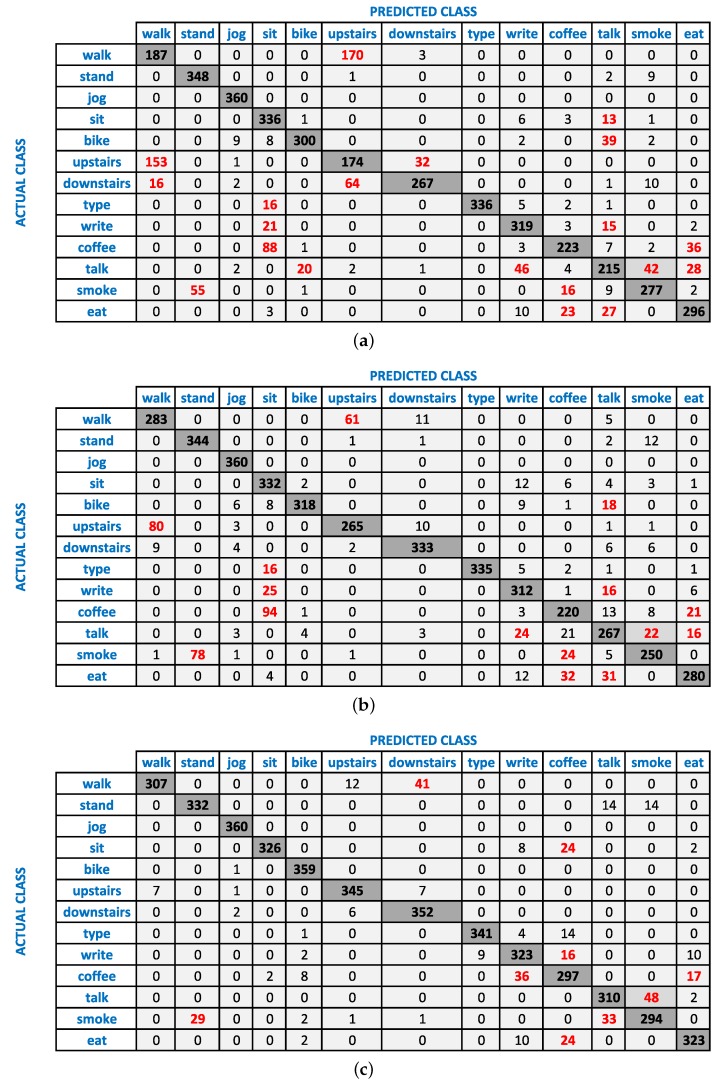
The confusion matrices of Naive Bayes classifier at a 5-s window size for; (**a**) $W_{A}$ (accelerometer at wrist); (**b**) $W_{AG}$ (accelerometer + gyroscope at wrist); (**c**) $WP_{AG}$ (accelerometer + gyroscope from both wrist and pocket). The major confused classes are shown in red.

**Figure 3:**
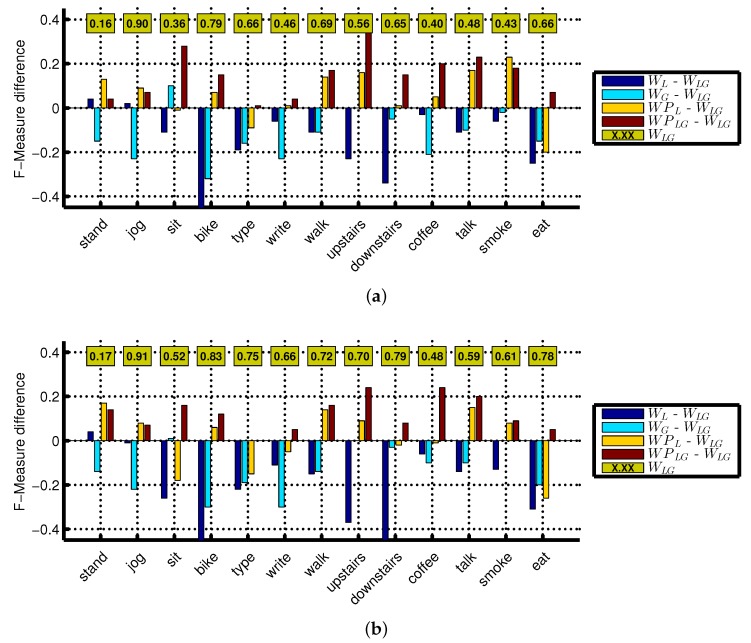
F-measure difference with respect to the reference ($W_{LG}$) for all activities using Naive Bayes classifier: (**a**) 2-s window; (**b**) 5-s window. The reference ($W_{LG}$) F-measure is shown in the boxes.

Previously, we have shown that the accelerometer and gyroscope complement each other for various activities at various single body positions [12,24]. For example, sitting and standing are better recognized by the accelerometer, while walking upstairs and downstairs are better recognized by the gyroscope. Their combination recognizes these activities with higher accuracy, thereby complementing each other (compensating for the weaknesses of either sensor). We also observe similar behavior in this work. As shown in Figure 1, these two sensors complement each other at the wrist position ($W_{AG}$) for various activities and further increase the recognition performance of walking, walking upstairs, walking downstairs and giving a talk. We show this behavior using confusion matrices of $W_{A}$ and $W_{AG}$ at a 5-s window in Figure 2a,b, respectively. It can be seen from these confusion matrices that the addition of the gyroscope to the accelerometer leads to better recognition results, specifically for using stairs and walking. Among these two sensors, the accelerometer performs better than the gyroscope on average for all activities, except walking, walking upstairs and walking downstairs. Similarly, the linear acceleration sensor and gyroscope complement each other to improve the recognition of various activities at the wrist ($W_{LG}$), as shown in Figure 3. However, we observe these improvements for more activities in $W_{LG}$ (linear acceleration sensor + gyroscope at wrist) than in $W_{AG}$ (accelerometer + gyroscope at wrist). One of the possible reasons is that there is more room for possible improvement in $W_{LG}$ compared to $W_{AG}$, because the recognition performance of various activities using the linear acceleration sensor (no gravity component) is lower than that of the accelerometer. In general, combining gyroscope data with the accelerometer or linear acceleration sensor improves the recognition performance of certain specific activities.

It is important to note that these sensor combinations do not always improve recognition performance. It can affect the recognition performance in three ways: increase, no change or decrease. In most cases, we observe an increase or no change for the combination of accelerometer and gyroscope. It is important to note that in the case of no change, the recognition is equal to the maximum of the two sensors. This means that such a combination still compensates for the weaknesses of either sensor in recognizing various activities. We observe a decrease in recognition performance with respect to the reference, if one of the sensors has very low recognition performance. For example, in Figure 1a, we see an 11% drop for smoking in $W_{AG}$ (accelerometer + gyroscope at wrist) with respect to $W_{A}$ (only accelerometer at wrist) at the 2-s window, because the recognition performance of the gyroscope for this activity is very low: only 40%. This affects the accelerometer recognition of 61%, thereby reducing the overall F-measure of their combination by 11%. A similar behavior can be seen for smoking activity at the 5-s window, as shown in Figure 1b; however, we see a relatively smaller drop here due to the larger window size. The respective confusion matrices of $W_{A}$ and $W_{AG}$ in Figure 2 show that the smoking while standing activity is mainly confused with standing activity.

As a next step, we combined motion data from the pocket position with wrist for better recognition of an extended set of activities. In general, the combination of accelerometer and gyroscope at the wrist and pocket positions ($WP_{AG}$, $WP_{A}$) improves the recognition of various activities, especially complex ones, as shown in Figure 1. For example, using a window of 2 s, $WP_{AG}$ (accelerometer + gyroscope from both wrist and pocket positions) improves the recognition of walking upstairs, walking downstairs, drinking coffee, giving a talk, smoking and eating by 29%, 4%, 17%, 16%, 22% and 13%, respectively, with respect to $W_{AG}$ (accelerometer + gyroscope at wrist). These improvements can be seen at the 5-s window in Figure 1 and also from the relative confusion matrices in Figure 2. We observe such improvement due to the additional context information from the pocket position. On the other hand, performance improvements due to such combinations on simple activities are lower, because usually, their reference performances are already high using single sensors, as shown in Figure 1. For example, we do not observe any important improvements for jogging, biking and typing, because they already have a high F-measure of 96%, 93% and 96%, respectively, at the wrist position alone using a window size of 2 s. However, there are some exceptions, as well. For example, in some cases, if the reference performance of these simple activities is low, then combining the sensor from both positions can improve their recognition performance. Walking is a simple activity; however, it is confused with walking upstairs and downstairs by the classifiers, as shown in Figure 2, thereby recognizing it with a relatively low F-measure of 70% (by Naive Bayes) at the 2-s window ($W_{AG}$: accelerometer + gyroscope at wrist). As in this case, its reference performance is relatively low; $WP_{AG}$ (accelerometer + gyroscope from both wrist and pocket positions) improves its recognition performance by 21%. As shown in Figure 1, $WP_{A}$ (accelerometer from both wrist and pocket positions) behaves in a similar way as $WP_{AG}$, except for the walking, walking upstairs and walking downstairs. For these three activities, the accelerometer data from both positions perform poorly. We had shown previously that the accelerometer performs poorly at recognizing walking upstairs and downstairs and needs additional gyroscope data for better recognition of these two activities [12]. This shows that it is better to use $WP_{AG}$ or $WP_{A}$ instead of $W_{AG}$ for recognizing complex activities, though it depends on the set of activities. If walking upstairs and walking downstairs is not part of the activity set, using $WP_{A}$ may be a better option than $WP_{AG}$ because of less energy consumption in $WP_{A}$.

Similarly, we observe improvements in $WP_{LG}$ (linear acceleration sensor + gyroscope from both wrist and pocket positions) mainly for complex activities, as shown in Figure 3. In general, $WP_{LG}$ follows the same trends (increase, decrease, no change with respect to its reference) as in $WP_{AG}$ (accelerometer + gyroscope from both wrist and pocket positions) and outperforms $W_{LG}$ (accelerometer + gyroscope at wrist). However, $WP_{LG}$ also improves the recognition performance of some simple activities, such as biking, walking, writing and typing. This is mainly because the reference performance for these activities is not very high, thereby leaving more room for possible improvement. Moreover, we argued in Section 1 that the recognition of biking can be better recognized at the pocket position, so combining the pocket with the wrist position may further improve the recognition of this activity. We observe this in $WP_{LG}$, where the recognition of biking is improved by adding motion data from the pocket position with the wrist as shown in Figure 3. Though we observe improvements for biking in $WP_{AG}$, they are relatively small, because the reference performance for biking is higher.

In general, the recognition performance using an accelerometer is higher than the performance of using a gyroscope and a linear acceleration sensor. For this reason, the absolute F-measure values of various activities are higher for $WP_{AG}$ (accelerometer + gyroscope from both wrist and pocket positions) than $WP_{LG}$ (linear acceleration sensor + gyroscope from both wrist and pocket positions). Moreover, the linear acceleration sensor and gyroscope cannot differentiate between sitting and standing. However, the recognition performance for the gyroscope and linear acceleration sensor can be improved in different ways; e.g., by optimizing the classifier’s parameters, using complex classifiers or additional features. We discuss some of these options in Section 4.4. If sitting and standing are not important for an application, then it may be a good idea to use the combination of the linear acceleration sensor with the gyroscope instead of the accelerometer for more generalized classification results.

Though we evaluated seven window sizes, we only presented results for 2 and 5 s in Figure 1 and Figure 3, because we observe similar trends for other window sizes in terms of performance improvements. However, the performance improvement due to combining motion sensors from both positions for complex activities becomes smaller with increasing window size. This happens because the reference performance increases at larger window sizes, thereby leaving less room for possible improvement. For example, such performance increase for smoking is 22% (reference: 51%), 4% (reference: 85%) and 0% (reference: 95%) at a window size of 2, 10 and 30 s, respectively. We observe a similar window size effect for scenarios involving the linear acceleration sensor. For better activity recognition of complex activities, a sensor combination at a bigger window size should be used, as we discuss in the next section.

Like the Naive Bayes classifier, we observe similar trends for the decision tree and KNN classifiers, as well. These results are presented in Appendix A.

### 4.2. The Effect of Window Size on Recognition Performance

**Figure 4:**
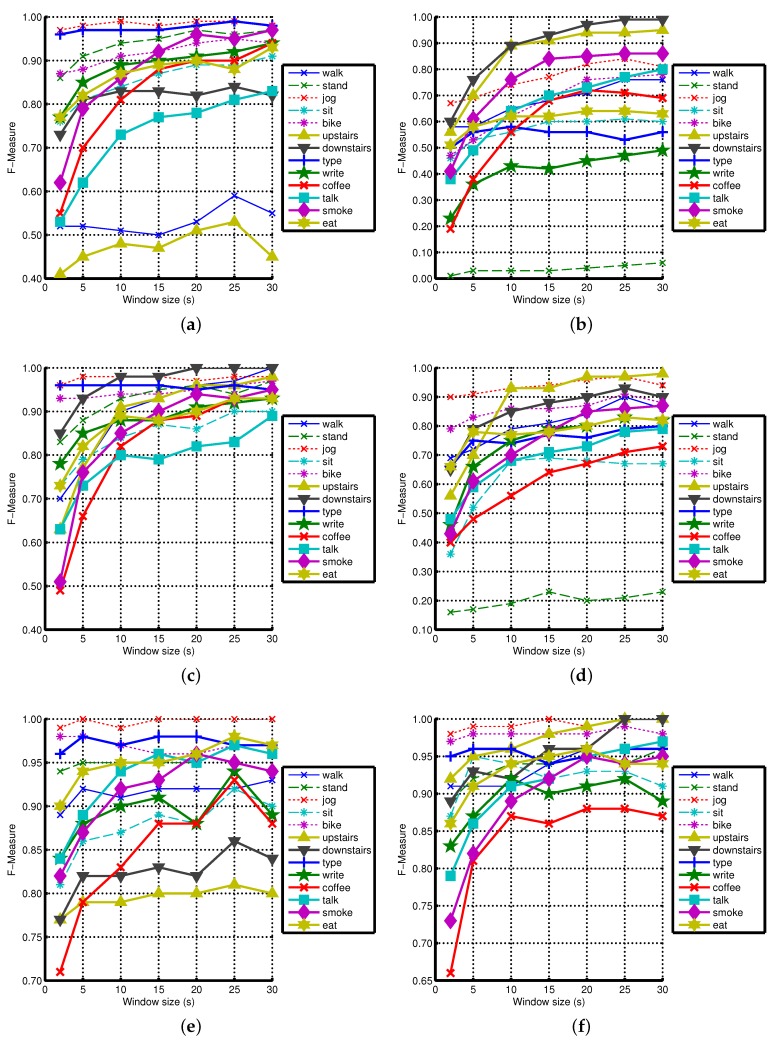
The effect of increasing window size on various activities using Naive Bayes classifier: (**a**) $W_{A}$; (**b**) $W_{G}$; (**c**) $W_{AG}$; (**d**) $W_{LG}$; (**e**) $WP_{A}$; (**f**) $WP_{AG}$.

In this section, we discuss the role of window size in the recognition of various activities. It has been shown before that a window size of 2 s is enough for recognizing the most common physical activities, such as walking, jogging, biking, sitting and standing, because they are repetitive [10,12]. However, we believe such a small window may not be enough to capture the repetitive pattern of relatively complex activities, such as smoking, eating, giving a talk and drinking coffee. Therefore, we evaluate the effect of varying the window size (2, 5, 10, 15, 20, 25, 30 s) in various scenarios. We observe improvements in recognition performance with increasing window size, mainly for complex activities. The detailed effect of window size in various scenarios using the Naive Bayes classifier is shown in Figure 4.

**Figure 5:**
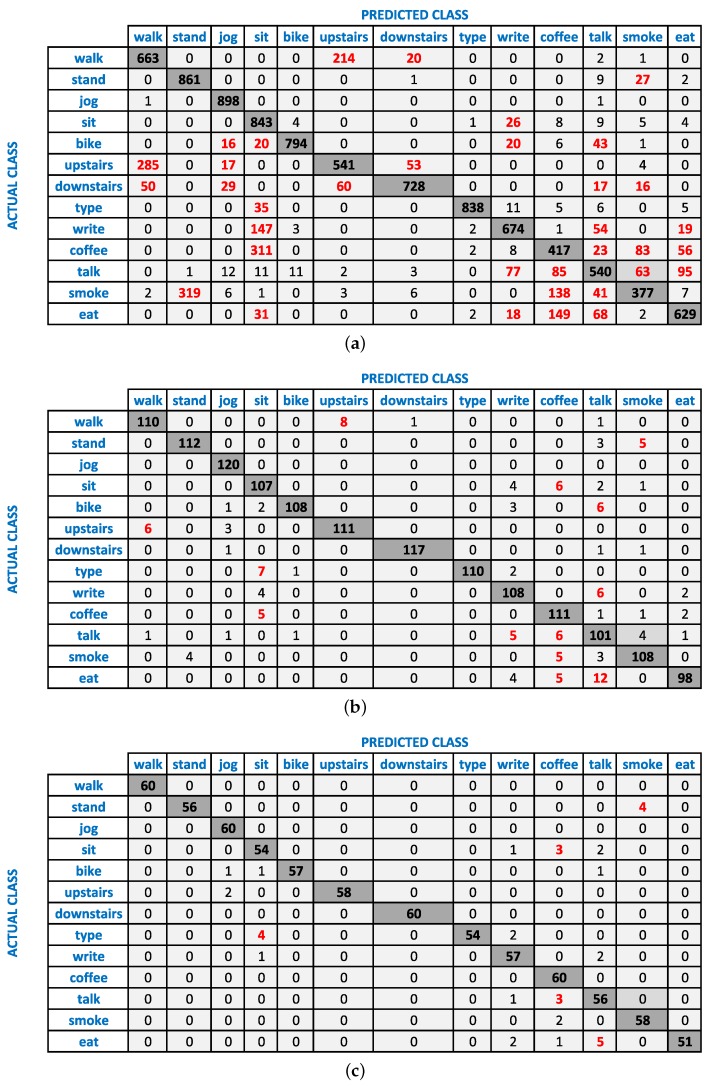
The effect of increasing window size for $W_{AG}$ using the Naive Bayes classifier at; (**a**) 2-s window; (**b**) 15-s window; (**c**) 30-s window. The major confused classes with each actual class are highlighted in red in these confusion matrices.

As shown in Figure 4, increasing the window size increases the recognition performance of less-repetitive activities involving hand gestures (smoking, eating, drinking coffee, giving a talk) in almost all scenarios. For example, the increase for smoking ranges up to 35%, 45%, 34%, 44%, 44%, 14%, 22% and 25% in $W_{A}$, $W_{G}$, $W_{L}$, $W_{AG}$, $W_{LG}$, $WP_{A}$, $WP_{AG}$ and $WP_{LG}$, respectively, which are important improvements. We observe similar improvements for eating, drinking coffee and giving a talk. We see such an increase for these complex activities because the classifiers need a larger window to learn the repetitive motion pattern for these activities. At smaller windows, these non-repetitive activities can be confused with the two postures: standing and sitting. For example, smoking while standing can be confused with standing, and eating or drinking while sitting can be confused with sitting. This can be seen from the confusion matrices of $W_{AG}$ in Figure 5. Moreover, these complex activities are confused with each other as well at smaller segmentation window sizes as shown in Figure 5. For example, smoking while standing and eating while sitting are confused with drinking coffee while sitting at the 2-s window. However, their recognition performance improves as we increase the window size from 2 to 15 and 30 s. In some cases, the performance improvements due to increasing window size become smaller, such as in $WP_{A}$, $WP_{AG}$ and $WP_{LG}$, because they already have high recognition performance at smaller windows, leaving less room for possible performance improvement. However, we still recommend using a larger window size for recognizing these less repetitive activities involving hand gestures, because these will be recognized in a better way.

In most cases using all of our three classifiers, we observe important improvements with increasing window size for walking, walking upstairs and downstairs, though these are very repetitive activities. During using stairs, there was a small walking activity on each floor to switch to the next floor (2–5 steps). This may confuse the classifier when we use a small window, because these steps are labeled as using stairs. However, this confusing effect may diminish with a larger window size. For these reasons, we need a larger window size for complex activities, even when a sensor combination is being used. It can be seen from the confusion matrices of $W_{AG}$ (accelerometer + gyroscope at wrist) in Figure 5 that these three activities are confused with each other at a smaller window size of 2 s. However, their performance improves with increasing window sizes, such as at 15 and 30-s windows.

The window size has little effect on the rest of the simple activities, because these activities are very repetitive in nature. For such activities, a recognizable motion pattern can be obtained in a shorter window of 2–5 s. Though we observe improvements for some of these activities with increasing window size, they are not consistent in all scenarios using all three classifiers. For example, the recognition of biking, writing and sitting is improved in some cases with increasing window size. However, in very few cases, increasing the window size causes a drop in the recognition performance of some simple activities. One of the possible reasons for such a drop could be random hand gestures by the participants during these activities. We use mean and standard deviation features, which can change with random hand gestures. For small windows, these random gestures are not a problem, as only a 2-s window of sitting is misclassified. However, for larger window sizes, it can erroneously classify a big chunk of data, such as 30 s of sitting, thereby leading to more loss in overall recognition performance. As we use two very basic features, we believe using additional features may help in countering random hand gestures with an increasing window size. We discuss a few possible optimizations in Section 4.4.

As for as the main observations regarding the effect of increasing window size on recognition performance are concerned, we observe similar behavior like Naive Bayes using KNN and decision tree classifiers. However, the absolute recognition performance values are different for these three classifiers. We have shown the effect of increasing window size on the recognition performance of various activities using KNN and decision tree in Appendix A.

### 4.3. Analysis Using Cross-Validation with Shuffling Data

As we pointed out earlier, we also evaluated all of the above scenarios using a stratified cross-validation with shuffling the data. This means that data from each participant was used in both training and testing. However, there is no overlap in the data used in these two sets. As we argued, recognition performance will be higher in such validation because it is more like a person-dependent validation. We observe the same, that the recognition performance is higher for all activities in almost all situations than in the validation method without shuffling the data. As far as the effect of increasing window size and sensor combination from pocket and wrist positions on the recognition performance of complex activities is concerned, we observe similar trends like the ones in the cross-validation without shuffling. However, the effect of increasing window size on simple activities is smaller than in Section 4.2. We observe small or no improvements for these simple activities with increasing window size, because their recognition performance is already high at small window sizes, thereby leaving less room for further improvements.

### 4.4. Optimizations for Recognizing Complex Activities

In the previous sections, we were only interested in comparative analysis of the effect of window size and sensor combination from two body positions on the recognition performance of various activities. Therefore, we did not optimize anything for higher recognition performance. There are a few scenarios where recognition performance (F-measure) was poor for complex activities, such as for $W_{L}$ (linear acceleration sensor at wrist), $W_{G}$ (gyroscope at wrist) and $W_{LG}$ (linear acceleration sensor + gyroscope at wrist). Using only the mean and standard deviation, the gyroscope and linear acceleration sensor perform poorly compared to the accelerometer, as shown in previous sections. It shows that these two features alone are not sufficient to differentiate between various activities for these two sensors. For example, the standard deviation or mean can be similar for similar activities, like smoking and eating; however, its minimum, maximum or frequency-based features may be different. Moreover, the median can counter the noise or random outliers unlike the mean. This can help in recognizing various activities at bigger window sizes. Hence, we extend our existing feature set with five more features: minimum, maximum, median, semi-quartile and the sum of the first ten FFT (Fast Fourier Transform) coefficients. These features have been shown to be suitable for running on mobile phones because of their low or medium computation and storage complexity [30].

**Figure 6:**
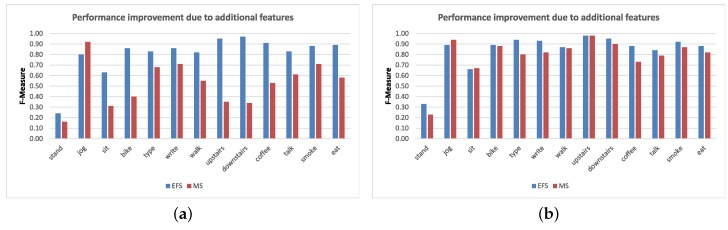
F-measure of various activities with Naive Bayes using only mean and standard deviation (MS) *vs*. our extended feature set (EFS) at 30-s window. (**a**) MS *vs.* EPS in $W_{L}$; (**b**) MS *vs.* EPS $W_{LG}$.

Using the Naive Bayes classifier, we first re-evaluated $W_{L}$ and $W_{G}$ with our new extended feature set at seven window sizes. However, we only observe important improvements for $W_{L}$ (linear acceleration sensor at wrist). Therefore, we only used extended features of the linear acceleration sensor in $W_{LG}$ (linear acceleration sensor + gyroscope at wrist) while only the mean and standard deviation for the gyroscope. There were important improvements for some complex activities in $W_{L}$ and $W_{LG}$. In recognition performance for all activities, we observed an average increase of 28% for $W_{L}$ and 5% for $W_{LG}$. These improvements are small for $W_{LG}$, because its recognition performance using the mean and standard deviation is not as small as for $W_{L}$, thereby leaving less room for further improvements. For example, in $W_{L}$, there was an increase of up to 31% for eating, 17% for smoking, 38% for drinking coffee, 22% for giving a talk and up to 63% for using stairs. On the other hand, such increases in $W_{LG}$ were up to 6% for eating, 5% for smoking, 21% for drinking coffee, 7% for giving a talk and 5% for using stairs. These improvements for $W_{LG}$ and $W_{L}$ at 30 s window can be seen in Figure 6. Though we do not show the results for smaller window sizes here, we observe increasing improvements in the recognition performance due to additional features with increasing window size. This behavior shows the importance of using a larger window size for complex human activities.

Our goal was to show that the recognition performance of these activities can be improved by selecting the right feature set. Though we explored a specific set of features, the selection of the right features in various scenarios can be further explored based on the type of activities that need to be recognized.

Some complex activities, such as smoking, eating, drinking coffee and giving a talk, are usually not as repetitive as walking or jogging. There can be small pauses between the actual hand gestures in these activities. We solve this problem to some extent by extending the window size. However, the window size can only be increased up until a certain limit. In addition to extending the window size, we propose to use a hierarchical approach where a rule-based mechanism is applied on top of a classifier output. For example, if there are a few smoking detections in a specific time frame, then that time frame as a whole can be re-classified as the smoking activity. Let us say that we store classification results for the last five minutes, while using a window size of 30 s. That means that we have 10 recent outputs at all times. We can apply a rule-based algorithm on these data, such as the example shown in Algorithm 1. We will explore and evaluate this further in our next study.

**Algorithm 1** Simple rule-based algorithm.
1:**procedure** MyProcedure   2:    **if** Classifier output is a complex activity A AND the number of its instances in the last 10 recent outputs >a specific threshold **then**   3:        Identify first instance of activity A.   4:        Classify outputs from the first instance till the last instance of A.   5:    **else**  6:        Retain the existing classification result.   


Even though a larger window size improves the recognition of complex activities, it can affect the recognition of other simple activities in a negative way, such as sitting and standing. One or two hand gestures can change the mean and standard deviation of our segmentation window so much that it can confuse the classifier. Such random hand gestures can lead to the wrong classification of either a small or big chunk of data depending on the window size. For example, such wrong classification will result in 30 s of wrongly-classified data for a window size of 30 s and 2 s for a window size of 2 s. This means that the size of the window has positive and negative effects depending on the type of activity. One solution to this problem is using different types of features that are less susceptible to such random gestures. Furthermore, this can be further improved by using adaptive windowing. For example, we can keep the window size at 2 or 5 s as a default. However, if we see an instance of complex activity, we increase the window size to, for example, 30 s for the next few minutes. If there is a complex activity going on, such as smoking or eating, which will last for a few minutes, then we can recognize it with the increased (larger) window size. If there is no complex activity going on and it is just a random hand gesture, we can revert back to the default smaller window size after some time. This idea needs to be further explored, which we intend to do in our next study. To recognize various simple and complex activities in a reliable way, a combination of additional features that counter random hand gestures, adaptive windowing and rule-based hierarchical classification can be used.

### 4.5. Limitations and Future Work

As our data collection was performed in a controlled environment, our results for complex activity recognition might be optimistic when compared to a real-world setup. However, it is the first step towards recognizing such activities in real-life scenarios. These activities can be performed in various ways. For example, eating activity can be comprised of eating a sandwich while walking, standing or sitting, eating soup while sitting or eating something with a knife and a fork in different postures. Moreover, eating in a group may lead to a different activity type if the participants are involved in a conversation. Such kinds of variations may result in different motion patterns. Similarly, drinking coffee or smoking in a group or in different postures can result in different patterns of motion data. Moreover, some people may smoke and drink coffee at the same time. The recognition of such complex activities is yet to be explored further; however, our work is an incremental contribution towards that direction. We plan to conduct more data collection experiments in real-life setups in the future.

At the time of data collection for this study, we did not have smart-watches, so we emulated them with a smartphone at the wrist position. As a smartphone is usually heavier than a smartwatch, using it at the wrist position may change the participant’s behavior; however, we did not explore that aspect in this study. Based on the observations, we expect no significant changes in the participants’ behavior while carrying out these activities. Currently, we are using a smartwatch (LG Watch R) for our next data collection experiment and plan to look at this aspect. Moreover, we also did not explore the energy efficiency aspects of the smartwatches, as it was not the main focus of the study. As discussed in [44], smartwatches have limited processing power and limited battery capacity, which may limit the use of applications that are continuously using sensor data in real time. This needs further exploration, though initial work towards energy-efficient context sensing has been done in [44], which also highlights some of the other challenges in using smartwatches for activity recognition.

## 5. Conclusions

At seven window sizes, we evaluated the effect of combining motion sensors at both the wrist and pocket positions for recognizing thirteen activities. For this purpose, we employed stratified cross-validation with and without shuffling the data. We showed that the sensor combination at both of these positions improves the recognition performance of various activities, especially complex ones, and outperforms the wrist position alone in most cases. However, these improvements are mainly observed at smaller window sizes, because the recognition of these complex activities is improved with increasing window size, thereby leaving less room for further improvement. In general, the recognition performance of smoking, drinking coffee, eating and giving a talk is increased with increasing window size. We observe similar trends for walking and using stairs. However, only increasing the window is not enough for these three activities, because the main increase in their recognition performance comes from the addition of the gyroscope with the accelerometer, either at the wrist or both wrist and pocket positions. For the rest of the simple activities, we observed relatively smaller improvements due to combining data from both pocket and wrist positions. For such a combination, we observe no or little effect on those activities where they have very high reference recognition performance. Though increasing the window size improved the recognition performance of various complex activities, it has a smaller effect on simple activities in most cases. However, we see improvements due to increasing window size for simpler activities when their reference performances are low. Though the sensor combinations improve the recognition of complex activities at smaller window sizes, we still recommend using a bigger window size for their reliable recognition. We also showed that the recognition of complex activities can be further improved with a careful selection of additional data features. We argue that both simple and complex activities can be recognized in a reliable way by using hierarchical classification and adaptive windowing. However, this needs to be further explored, which we plan to cover in our future work. We also plan to collect more data for a further extended set of activities and recognize these activities using a state-transition-based algorithm, such as the Hidden Markov Model (HMM).

## Figures and Tables

**Figure A1:**
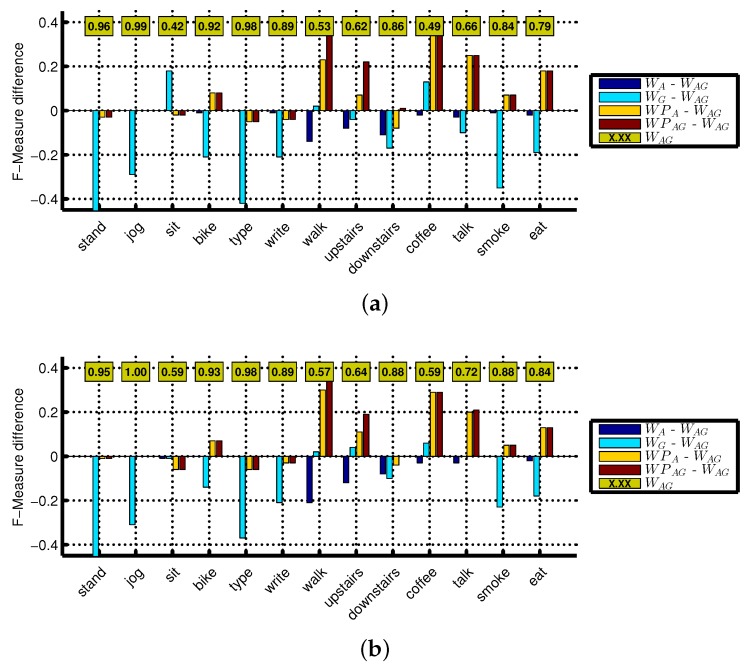
F-measure difference with respect to the reference ($W_{AG}$) for all activities using KNN: (**a**) 2-s window; (**b**) 5-s window. The reference F-measure is shown in the boxes.

**Figure A2:**
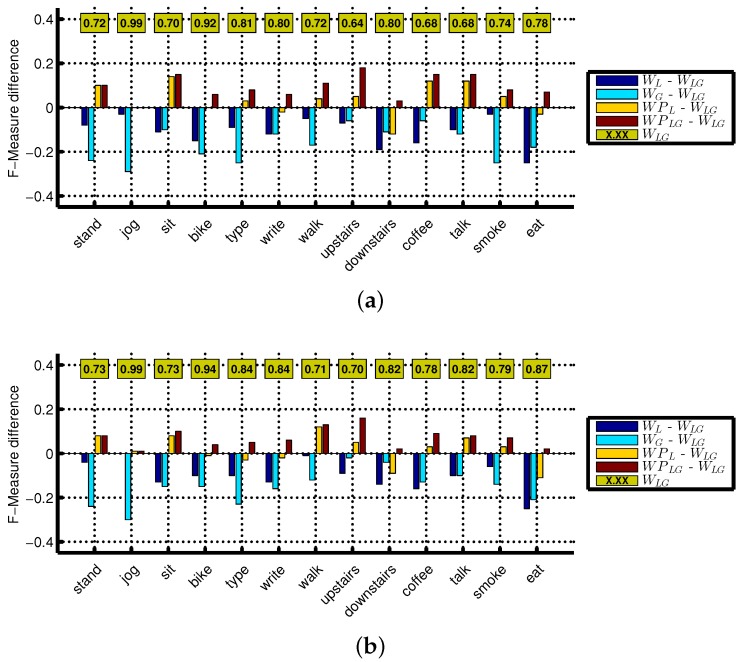
F-measure difference with respect to the reference ($W_{LG}$) for all activities using KNN: (**a**) 2-s window; (**b**) 5-s window. The reference F-measure is shown in the boxes.

**Figure A3:**
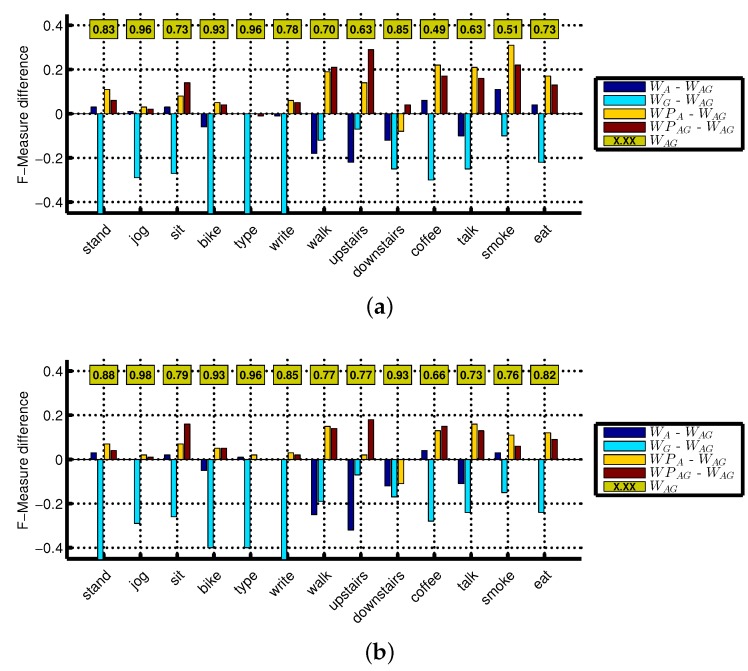
F-measure difference with respect to the reference ($W_{AG}$) for all activities using decision tree: (**a**) 2-s window; (**b**) 5-s window. The reference F-measure is shown in the boxes.

**Figure A4:**
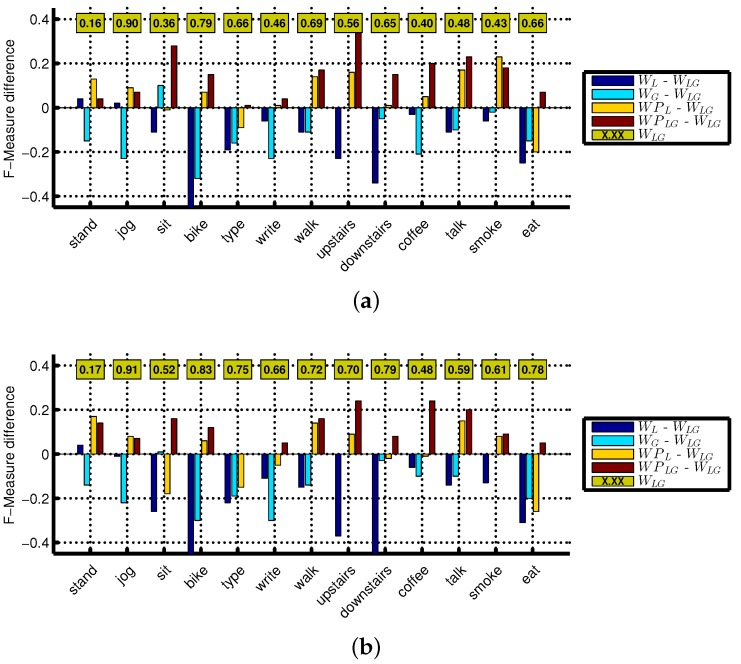
F-measure difference with respect to the reference ($W_{LG}$) for all activities using decision tree: (**a**) 2-s window; (**b**) 5-s window. The reference F-measure is shown in the boxes.

**Figure A5:**
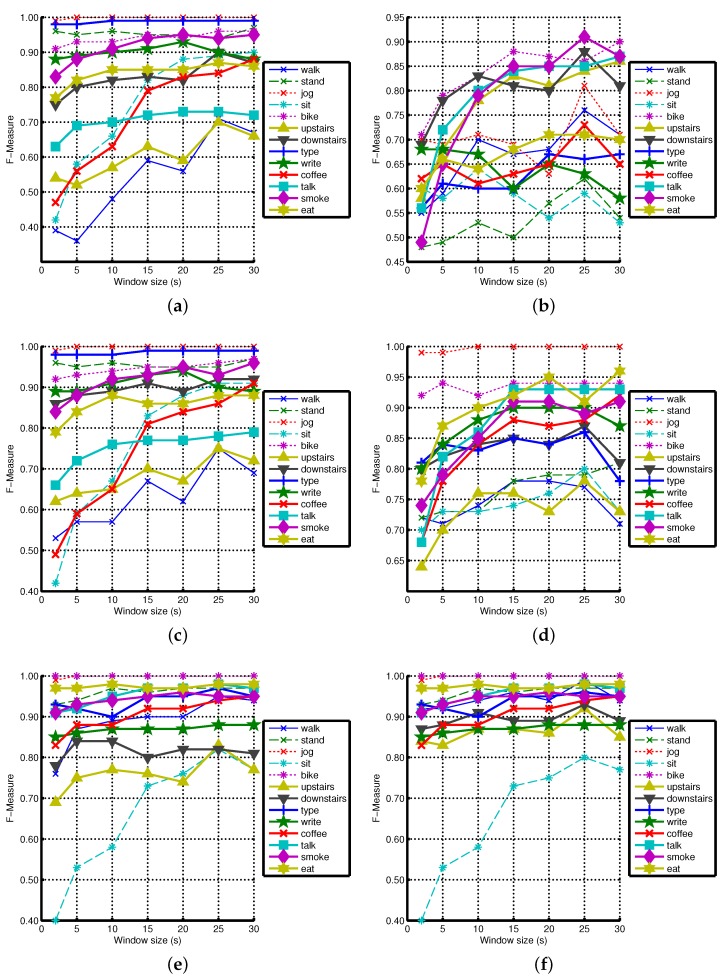
The effect of increasing window size on various activities using KNN. (**a**) $W_{A}$; (**b**) $W_{G}$; (**c**) $W_{AG}$; (**d**) $W_{LG}$; (**e**) $WP_{A}$; (**f**) $WP_{AG}$.

**Figure A6:**
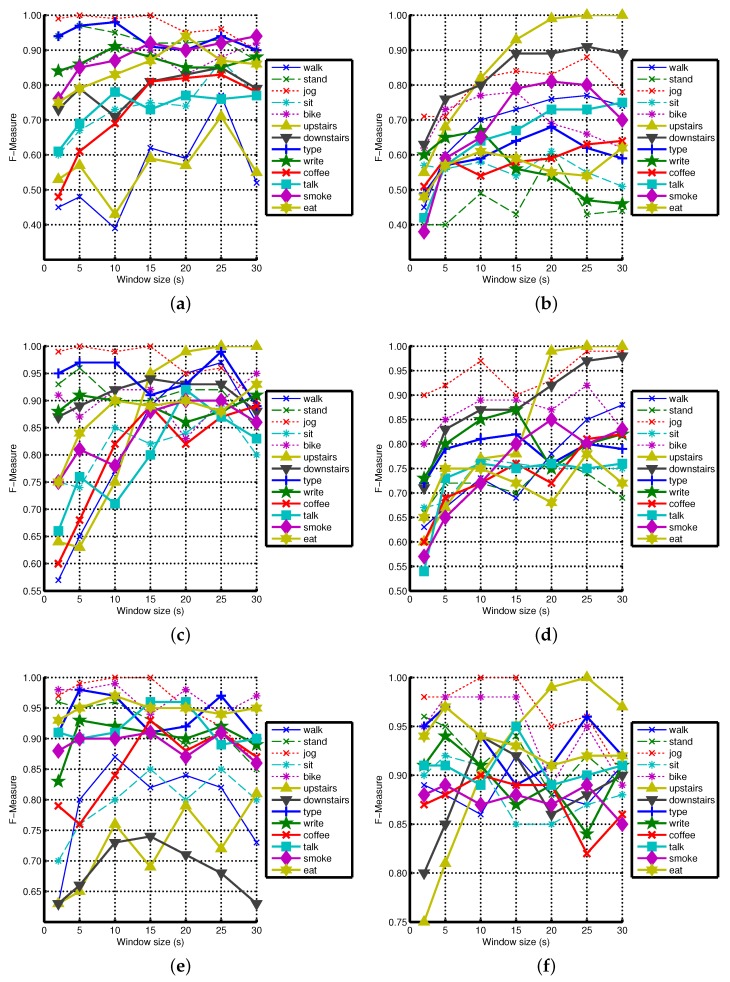
The effect of increasing window size on various activities using decision tree. (**a**) $W_{A}$; (**b**) $W_{G}$; (**c**) $W_{AG}$; (**d**) $W_{LG}$; (**e**) $WP_{A}$; (**f**) $WP_{AG}$.

## Appendix A. Classification Results of KNN and Decision Tree

In this Appendix, we describe the parameter settings of the classifiers used in our analysis, such as KNN, decision tree and Naive Bayes. Moreover, we present the results of KNN and decision tree classifiers in various scenarios. For each of these two classifiers, we show its classification results in evaluating the effect of sensor combinations and increasing the window size on the recognition performance.

### Appendix A.1. Classifiers Settings for Its Parameters

We used Scikit-learn for our data analysis, which is a Python based toolkit. We used these classifiers in their default settings with a few changes, as shown in their respective Python calls. For their default settings, readers are suggested to go to the Scikit-learn website [34].

**Naive Bayes:** This implements the Gaussian Naive Bayes algorithm for classification. We used it in its default settings, and its respective Python call in Scikit-learn is as follows:

modelnaivebayes = GaussianNB()

**K-Nearest Neighbor:** This implements the k-nearest neighbors’ vote-based classifier. The Python call for this classifier with its relative parameters is as follows:

modelknn = KNeighborsClassifier(n_neighbors = 3, algorithm = ‘ball_tree’, p = 2,

metric = ‘minkowski’)

**Decision Tree:** This implements an optimized version of the CART algorithm. The Python call for this classifier is as follows:

modeldecisiontree = DecisionTreeClassifier(random_state = 1)

### Appendix A.2. The Effect of Sensors Combination on Recognition Performance

This section presents the effect of sensor combinations on the recognition performance of various activities using decision tree and KNN classifiers. The classification results of the KNN classifier for various senor combinations are shown in Figure A1 and Figure A2, whereas those of the decision tree classifier are shown in Figure A3 and Figure A4.

### Appendix A.3. The Effect of Window Size on Recognition Performance

This section presents the effect of increasing window size on the recognition performance of various activities using the decision tree and KNN classifiers. These results are shown in Figure A5 for KNN and in Figure A6 for decision tree.
